# Evaluating HIV Rapid/Point of Care Testing among Risk Factor Groups in Ontario, 2011 to 2018

**DOI:** 10.1186/s12889-022-14939-3

**Published:** 2023-01-24

**Authors:** Heather Rilkoff, Hadia Hussain, Juan Liu, Ken English, Joanne Lush, Ashleigh Sullivan, Vanessa Tran, Vanessa Allen, Michelle Murti

**Affiliations:** 1 Public Health Ontario, 661University Ave Toronto, Toronto, ON Canada; 2 AIDS and Hepatitis C Bureau, Ontario Ministry of Health and Long-Term Care, Toronto, Canada

**Keywords:** HIV, Point-of-Care testing, Test performance

## Abstract

**Objectives:**

In 2014, Ontario’s Point-of-Care (POC) test providers were advised to focus efforts on provincially defined priority populations who experience a greater risk of HIV. Our objective was to describe the POC program before, during and after this change, including tester characteristics, follow-up testing results, positive predictive value (PPV) over time, and trends and characteristics of those with reactive test results without a confirmatory serological specimen.

**Methods:**

Test-level data of POC screening and confirmatory results were extracted from the Public Health Ontario HIV Datamart. Final test results were defined based on results of the confirmatory blood sample, or the POC test for “non-reactive” tests. Testing volumes, percent of total tests, percent positivity and PPV were calculated overall, annually, and by exposure group.

**Results:**

Overall testing volumes decreased by 39.8% between 2014 and 2018. The majority of confirmed positive tests were in the men who have sex with men (MSM) exposure category, followed by HIV-endemic and heterosexual – no identified risk (heterosexual—NIR). Overall percent positivity decreased from 0.59% in 2011 to 0.42% in 2015 (change of 0.17%, 95% CI 0.03% to 0.31%), increasing to 0.69% in 2018 (change of 0.27%, 95% CI 0.20% to 0.34%). Increases in percent positivity corresponded with a decrease in the overall proportion of tests conducted in low-risk populations. When compared to the heterosexual-NIR category, PPV was significantly higher for men who have sex with men – people who use injection drugs (MSM-PWID) (52.7% compared to 100%, *P* < .001), MSM (52.7% compared to 95.4%, *P* < .001), HIV-endemic (52.7% compared to 91.5%, *P* < .001), heterosexual – partner with identified risk (heterosexual—PIR) (52.7% compared to 77.3%, *P* = .042), and people who use injection drugs (PWID) (52.7% compared to 81.3%, *P* = 0.007). A total of 13.5% of reactive POC results did not have a serological sample submitted.

**Conclusions:**

Targeted testing towards populations at higher risk of HIV improved the overall test performance characteristics of Ontario’s POC testing program. While not unexpected, the large discrepancies between PPV in higher-risk, compared to lower-risk populations, suggests the need for greater awareness and messaging of the likelihood of false positive test results in different populations.

## Introduction

The INSTI HIV-1/HIV-2 Rapid Antibody Test (bioLytical Laboratories) provides rapid point-of-care (POC) testing for HIV and has been approved and available in Canada since 2005 [1]. POC testing programs in a variety of settings have been shown to be acceptable, feasible and an important engagement tool for testing and linkage to care [2]. Models of delivering POC testing include primary care, STI clinics, community-based organizations and emergency departments [2], and have more recently expanded to pharmacies [3]. Additionally, home-based POC testing was licensed for use and sale in Canada in November 2020 [4].

Despite the known benefits of POC testing, an ongoing concern of POC testing in Canada is the test performance characteristics compared to conventional serological testing for low-prevalence populations [5]. For example, a recent scoping review of POC testing in different settings in Canada indicated that the percentage of reactive POC tests that were false positive varied from 4 to 33% [5]. Manufacturer reported sensitivity and specificity of the INSTI POC test is at 99% or greater [6]. However, given the test characteristics, and the seriousness of an HIV diagnosis, in Canada, reactive POC results require follow-up confirmatory serological testing, with linkage to care ideally initiated at the time of the reactive POC result [1]. With the expansion of POC testing to populations with lower risk and a lower prevalence for HIV, there is an increasing likelihood of false positive results [7]. For lower risk populations receiving a reactive POC result, there is an opportunity to provide counselling and management of the possibility of a false positive result in the interim until a more definitive test result is available.

In Ontario, Canada, a publicly-funded program for HIV POC testing has been available since 2007 [8]. The majority of these tests are offered anonymously, but also include nominal and coded tests [9]. Since 2009, POC testing guidance was issued to harmonize the training, quality assurance, counselling and follow-up expectations for all providers offering POC testing in the province [10]. In addition to the volume of POC testing offered, a unique feature of the Ontario POC program is the collection of data from the usual HIV laboratory requisition form from all testers, regardless of POC test result, including their reason for testing and demographic information. The robust data collection of those accessing POC testing, as well as linkage to their subsequent serological test results for those who test positive or may be in the ‘window period’ at the time of POC testing, enables assessment of field test performance with a large-scale program. This includes patients who may refuse to submit a serological specimen despite receiving a reactive test result, who are otherwise not captured in traditional surveillance data in the absence of laboratory confirmation.

Beginning in 2014, POC test providers in Ontario were advised to focus POC testing efforts towards provincially defined priority populations who experience a greater risk of HIV [11]. These include men who have sex with men (MSM), people who use injection drugs (PWID), Indigenous populations, African, Caribbean and Black populations, and at-risk women. Our objective was to describe the POC program, tester characteristics, and follow-up testing results in the time span over this change, from 2011 to 2018, including positive predictive value (PPV) over time, and trends and characteristics of those with reactive test results without a confirmatory serological specimen.

## Methods

### Data source

Test-level POC data were extracted from the Public Health Ontario HIV Datamart, for the POC test requisition data and the clinical laboratory data of confirmatory samples linked to reactive or window period POC tests. Test requisition data includes age, sex, reason for testing, and testing provider (local public health unit, clinic or community-based health centre). Data was restricted to 2011 to 2018 due to data quality issues that affected the reliability of POC data capture prior to 2011 and extracted on September 18, 2019. Results for patients younger than 15 years of age were excluded from the analysis.

### Classification of exposure category

Clinician entered risk factor information from the HIV test requisition was used to create hierarchical and mutually exclusive exposure categories for each patient, representing their most likely source of HIV risk [12]. These exposure categories were used to approximate Ontario’s priority population groups. Data on specific priority population groups (i.e., race, ethnicity and Indigenous status), was not collected on test requisition forms in that time period.

Table 1 describes exposure categories included in the analysis, listed in hierarchical order. All analyses included those with no identified risk factors and those with unknown or missing information. Exposure categories clotting factor, blood products, and mother to child transmission (*n* = 13) were excluded from this analysis.

**Table 1:** HIV exposure categories, in hierarchical order

Exposure category	Risk factors
Men who have sex with men and who use injection drugs (MSM-PWID)	Males who use injection drugs and have male sexual partner
Men who have sex with men (MSM)	Males with male sexual partner
People who use injection drugs (PWID)	People who use injection drugs
HIV-endemic	Having lived in a country where the prevalence of HIV among adults (15–49 years old) is 1.0% or greater and one of the following criteria is met: at least 50% are attributed to heterosexual transmission; a male to female ratio of 2:1 or less among prevalent infections; or HIV prevalence greater than or equal to 2% among women receiving prenatal care
Heterosexual – partner with identified risk (PIR)	Being male or female and indicating sex with a person of the opposite sex/gender who is either HIV-positive, a person at risk of HIV, a person who uses injection drugs, from an HIV-endemic area, had a blood or clotting factor transfusion, or is bisexual
Heterosexual – partner with no identified risk (NIR)	Being male or female and indicating sex with a person of the opposite sex/gender who has no identified risk
No identified risk	Indicating “none” or “other” or “needlestick injury” as a risk factor
Unknown/missing	No risk factors indicated (form not completed)

### Classification of POC results and Confirmatory Test Results

POC provider entered POC test results on the test requisition were extracted from the datamart: “reactive” (POC test implies virus may be present, and confirmatory laboratory testing needed), “non-reactive” (POC test implies virus is not present, no additional laboratory testing needed), or “non-reactive—window period”. Tests were considered to be in the “window period” if the test was performed within 90 days of possible exposure to HIV [1]. “Reactive” and “non-reactive—window period” results were assessed for linkage to a confirmatory serological test result associated with the test requisition.

We defined the final test result based on results of the confirmatory blood sample for “reactive” and “non-reactive – window period” tests, and the POC test for “non-reactive” tests. Tests with no confirmatory sample sent were classified as having a negative final result, as per Ontario surveillance guidelines [9].

### Calculation of measures

Testing volumes, percent of total tests, and percent positivity were calculated overall, annually, and by exposure group using all test results as the denominator, and all first-time positive test results as the numerator. Tests that were identified in the HIV Datamart as repeat positives, based on patient data linkage, were excluded from the calculation of percent positivity.

Positive predictive value (PPV) was calculated overall, annually, and by exposure group, using all POC reactive samples with a blood sample sent. PPV was calculated as the percent of POC reactive tests that were confirmed positive by laboratory testing. As the primary focus of this analysis was on the performance of the INSTI test as delivered by the Ontario POC program, both first time and repeat positive test results were included in the analysis.

Changes over time for percent positivity and PPV were assessed for statistical significance by calculating confidence intervals using the standard error of the difference in percentage between the time frame. Statistically significant difference in PPV between exposure groups was calculated using Fisher’s exact test with Bonferroni correction. All statistical analyses were performed using Stata 15.1.

## Results

### Test volumes and characteristics of POC testers and sites

POC testing numbers in Ontario increased from 25,901 in 2011, to a peak of 30,103 in 2014, and subsequently declined each year to a low of 18,128 in 2018. The majority of POC tests were conducted in males (ranging from 64.6% of all tests in 2011 to 76.8% in 2018), the 20–29 year age group (ranging from 47.6% of all tests in 2011 to 39.9% in 2018), and anonymously (ranging from 59.9% of all tests in 2011 to 72.6% in 2018). Tester proportions by exposure category shifted over time, with testers in the MSM exposure category increasing from 29.0% of tests in 2011 to 55.7% of tests in 2018, and testers in the heterosexual—NIR category declining from 58.8% of tests in 2011 to 29.9% of tests in 2018.

**Fig. 1:**
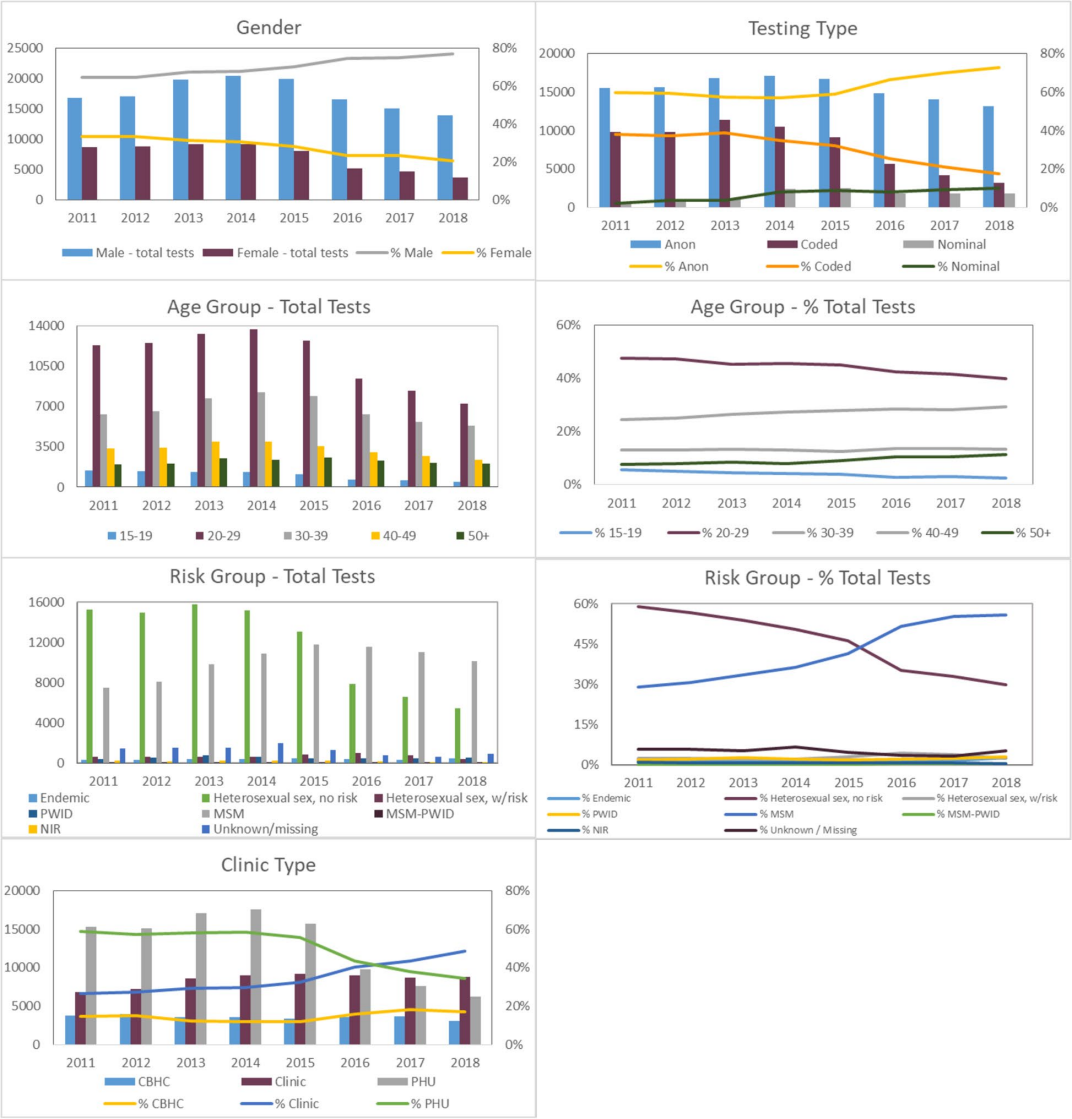
Demographics of POC testing participants, 2011–2018

Overall testing volumes decreased by 39.8% between 2014 and 2018, with larger decreases for females (59.6%), testers ages 15–19 (64.5%), testers ages 20–29 (47.3%), as well as for coded tests (69.8%) and tests conducted at local public health unit clinics (64.6%) (Fig 1). Decreases in testing volumes in these groups were largely the result of a decrease in testing in the heterosexual—NIR exposure category, which declined by 64.3% between 2014 and 2018, and represented 86.9% of the decrease in tests in females, and 79.8% of the decrease in tests in ages 20–29.

### Testing results and test-performance characteristics

There were 1,348 reactive and 9,470 window period tests between 2011 and 2018. A total of 1,077 tests (10.0% of all reactive and window period tests) were confirmed positive after an initial POC reactive or window period test, averaging 135 tests per year. The majority of confirmed positive tests were in the MSM exposure category (79.6%), followed by HIV-endemic (6.6%), heterosexual—NIR (4.2%), PWID (3.3%), MSM-PWID (3.0%), and heterosexual—PIR (2.8%).

**Fig. 2:**
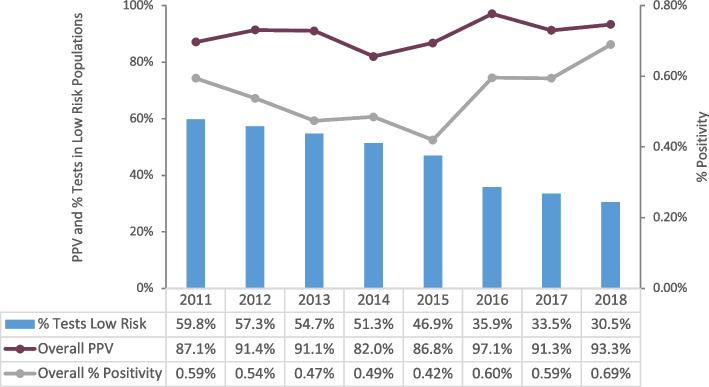
Percent of tests in low-risk populations, overall PPV, and overall percent positivity, by year

Overall percent positivity decreased from 0.59% in 2011 to 0.42% in 2015 (difference in percent 2011 to 2015 = 0.17%, 95% CI 0.03% to 0.31%), increasing to 0.69% in 2018 (difference in percent 2015 to 2018 = 0.27%, 95% CI 0.20% to 0.34%). Increases in positivity rates corresponded with a decrease in the overall proportion of tests conducted in low-risk populations (defined as testers indicating no identified risk (NIR) and testers indicating heterosexual sex with partners with no identified risk (heterosexual-NIR) – see Table 1 for definitions). Overall PPV fluctuated over time; there was a non-significant decrease from 87.1% in 2011 to 82.0% in 2014 (difference in percent 2011 to 2014 = 5.1%, 95% CI -2.9% to 13.1%), a significant increase to 97.1% in 2016 (difference in percent 2014 to 2016 = 15.1, 95% CI 8.3% to 21.9%), and a non-significant decrease to 93.3% in 2018 (difference in percent 2016 to 2018 = 3.8%, 95% CI -1.6% to 9.2%) (Fig 2).

PPV varied considerably by exposure category. When compared to the heterosexual-NIR category, PPV was significantly higher for the MSM-PWID (52.7% compared to 100%, *P* < 0.001), MSM (52.7% compared to 95.4%, *P* < 0.001), HIV-endemic (52.7% compared to 91.5%, *P* < 0.001), heterosexual – PIR (52.7% compared to 77.3%, *P* = 0.042), and PWID (52.7% compared to 81.3%, *P* = 0.007). While PPV remained consistently high over time in the MSM-PWID exposure categories and increased in the MSM and HIV-endemic exposure group after 2015, PPV fluctuated in other exposure categories and showed no consistent trend over time (Table 2).

**Table 2:** Total POC Tests, total reactive POC tests (with blood sample sent), and PPV, by exposure category, 2011 to 2018

		2011	2012	2013	2014	2015	2016	2017	2018	Total
HIV-endemic	Total POC Tests	305	349	415	443	495	398	299	475	3179
	Total reactive POC Tests	6	8	12	15	10	7	11	13	82
	PPV	100.0%	100.0%	91.7%	73.3%	80.0%	100.0%	100.0%	100.0%	91.5%
Heterosexual—NIR	Total POC Tests	15,235	14,961	15,811	15,203	13,080	7855	6588	5423	94,156
	Total reactive POC Tests	24	20	13	18	9	9	8	9	110
	PPV	37.5%	60.0%	46.2%	44.4%	44.4%	100.0%	62.5%	55.6%	52.7%
Heterosexual—PIR	Total POC Tests	627	669	626	628	875	1001	776	430	5632
	Total reactive POC Tests	6	10	4	5	7	5	5	2	44
	PPV	83.3%	70.0%	75.0%	80.0%	71.4%	100.0%	80.0%	50.0%	77.3%
PWID	Total POC Tests	446	548	798	645	505	499	462	549	4452
	Total reactive POC Tests	11	6	8	6	9	4	3	1	48
	PPV	81.8%	100.0%	75.0%	66.7%	100.0%	75.0%	33.3%	100.0%	81.3%
MSM	Total POC Tests	7522	8085	9828	10,912	11,770	11,529	11,058	10,101	80,805
	Total reactive POC Tests	132	109	113	129	107	107	92	104	893
	PPV	96.2%	98.2%	98.2%	89.1%	91.6%	98.1%	95.7%	97.1%	95.4%
MSM—PWID	Total POC Tests	52	65	67	61	59	71	67	78	520
	Total reactive POC Tests	5	7	5	4	1	2	5	4	33
	PPV	100.0%	100.0%	100.0%	100.0%	100.0%	100.0%	100.0%	100.0%	100.0%
NIR	Total POC Tests	246	170	231	240	223	147	126	102	1485
	Total reactive POC Tests	0	0	0	0	0	1	2	0	3
	PPV	-	-	-	-	-	100.00%	50.00%	-	66.7%
Unknown/missing	Total POC Tests	1468	1550	1547	1967	1334	813	646	970	10,295
	Total reactive POC Tests	2	2	2	1	1	2	0	2	12
	PPV	50.0%	50.0%	50.0%	0.0%	0.0%	50.0%	-	50.0%	41.7%

### Reactive tests missing a serological sample

A total of 13.5% (*n* = 182/1,348) of reactive POC results did not have a serological sample submitted. The number of reactive tests without a serological sample submitted fluctuated over time and did not show a consistent trend. Those with unknown or missing risk factors (35.3%), heterosexual – NIR (30.4%), PWID (25.0%) and NIR (25.0%) had the largest proportion of reactive tests where no confirmatory sample was submitted (Table 3).

**Table 3:** Total reactive tests (with and without blood sample), and total reactive tests with no blood sample sent, overall, by year and by exposure*

	Total reactive tests	No sample submitted	% No sample
Total	1348	182	13.5%
2011	201	23	11.4%
2012	172	20	11.6%
2013	174	25	14.4%
2014	202	31	15.4%
2015	163	27	16.6%
2016	139	11	7.9%
2017	135	14	10.4%
2018	162	31	19.1%
HIV-endemic	93	15	16.1%
Heterosexual–NIR	138	42	30.4%
Heterosexual-PIR	47	7	14.9%
PWID	60	15	25.0%
MSM	955	93	9.7%
MSM PWID	34	3	8.8%
NIR	4	1	25.0%
Unknown/missing	17	6	35.3%

^*^Excludes repeat positives

## Interpretation

Assessment of Ontario’s POC testing program, a high-volume system with robust linkages to epidemiological data on all testers, found significant shifts in populations tested after programmatic direction changes in 2014 to focus on priority populations. Analysis shows that the decreased proportion of tests among low prevalence populations had the anticipated outcome of decreasing the percentage of false positives in testers with low risk. Additionally, further analysis is warranted to investigate potential reasons/barriers for testers who have positive POC results without a confirmatory testing specimen, particularly for testers in the unknown and the heterosexual—NIR groups.

POC has been shown to be particularly beneficial to higher risk populations, with past research identifying increases in testing among previously untested individuals and greater knowledge of HIV status [13, 14]. In Ontario, the POC program had a higher positivity rate than traditional HIV testing, (0.69% compared to 0.12% in 2018), and a larger proportion of testers from higher risk groups (over two-thirds in POC, compared to approximately one-quarter in traditional testing in 2018) [9]. Previous studies have also described high levels of satisfaction among participants of HIV POC programs, who identified these interventions as more flexible, less invasive and less stressful than traditional HIV serological testing [5].

The majority of prior research on the benefits from POC testing have been among higher risk populations [5, 14]. Routine testing for HIV as part of screening for all adults has been touted as a mechanism to reach those who may not identify as having a risk factor and to de-stigmatize testing [15]. This analysis suggests caution is required for considering the use of POC testing, versus traditional testing, for broad population testing including those at lower risk of infection. False positives among those in the unknown and heterosexual- NIR groups, paired with higher rates of failing to submit a confirmatory specimen among testers in these groups may contribute to unfounded concern of a positive result in these populations. The results from this analysis of Ontario’s POC program suggest that caution is required when testing lower-risk populations in non-endemic settings, given PPV of 41.7% and 52.7% for those in the unknown risk and heterosexual – NIR groups, respectively. While the proportion of false-positive test results represent a very small fraction of the overall number of POC tests conducted annually, the variation in test performance between higher and lower-risk populations suggests the need for different approaches to pre and post counselling in persons without identified risk factors in order to avoid unnecessary concern and/or misinterpretation due to false positive test results. This includes emphasizing the importance of confirmatory testing, as this study noted that approximately 1/3 of persons without known risk factors for HIV with a reactive test result did not submit a follow-up serological specimen. Awareness around the potential for a false-positive test result may be particularly needed given the advent of HIV self-testing and the expansion of POC testing to different settings and provider types [16].

Overall PPV in this study was slightly lower in Ontario than in other Canadian studies [5], apart from one smaller study of at-risk populations reporting a PPV of 66.7% (4 out of 6 reactive tests confirmed positive) [17]. While none of the studies stratified PPV by exposure category, a study in Vancouver noted that PPV was lower in lower risk settings (Primary care and Public Health / STI Clinics) [18]. Additional studies of POC test performance that stratify by exposure or risk factor information may help provide insights into whether these differences are the result of different participant characteristics.

Limitations of this study include the use of a test-level dataset with a large proportion of anonymous tests (ranging from 59.9% of all tests in 2011 to 72.6% in 2018), that does not allow duplicate tests or prior positive results to be linked and accounted for at patient-level. Exposure information was missing for approximately 5% of tests; and entered exposure information may be misclassified due to inaccurate data collection or entry, or transcription error by the individual completing the requisition. Some of the exposure groups have a low number of reactive results due to either low testing numbers or very low prevalence of infection. Results for these populations, particularly trends over time, should be interpreted with caution due to low numbers. Additionally, test performance of individual POC sites were not assessed, and there is the potential for confounding to occur if false-positive test results were caused by testing errors at specific sites that also had larger patient populations of low-risk clients. Finally, as non-reactive tests that are not in the window period for potential infection were not subjected to confirmatory testing, other test performance characteristics (i.e., sensitivity, negative predictive value) could not be assessed.

## Conclusion

Targeted testing towards populations at higher risk of HIV improved the overall test performance characteristics of Ontario’s POC testing program. While not unexpected, the large discrepancies between PPV in higher-risk, compared to lower-risk populations, suggests the need for greater awareness and messaging of the likelihood of false positive test results in different populations. This may be particularly important as POC testing expands to different modalities and settings (e.g., self-testing), in order to prevent unnecessary concern or misinterpretation of HIV status.

## Data Availability

The full dataset supporting the conclusions of this article cannot be shared due to privacy concerns. Please contact the authors via Public Health Ontario (privacy@oahpp.ca) to discuss the feasibility of other requests for data from this manuscript.
